# Extra Virgin Olive Oil-Based Green Formulations With Promising Antimicrobial Activity Against Drug-Resistant Isolates

**DOI:** 10.3389/fphar.2022.885735

**Published:** 2022-04-25

**Authors:** Marisa Di Pietro, Simone Filardo, Roberto Mattioli, Antonio Francioso, Giammarco Raponi, Luciana Mosca, Rosa Sessa

**Affiliations:** ^1^ Department of Public Health and Infectious Diseases, Faculty of Pharmacy and Medicine, “Sapienza” University of Rome, Roma, Italy; ^2^ Department of Biochemical Sciences, Faculty of Pharmacy and Medicine, “Sapienza” University of Rome, Roma, Italy

**Keywords:** antimicrobial activity, extra virgin olive oil extract, oleocanthal, oleacein, NADES, drug-resistant isolates

## Abstract

Extra virgin olive oil (EVOO) from *Olea europaea* L. drupes, a cornerstone in the Mediterranean diet, is well known for its nutritional and health properties, especially for prevention of cardiovascular diseases and metabolic disorders. Traditionally, beneficial health effects have been largely attributed to the high concentration of monounsaturated fatty acids, and in recent years, these have also been related to other components including oleacein and oleocanthal. Here, we evaluated, for the first time, the antimicrobial activity of different green extra virgin olive oil-based formulations in natural deep eutectic solvents (NaDESs) emerging as powerful and biocompatible solvents. Specifically, the antimicrobial activity of the EVOO extract, as well as purified oleocanthal and oleacein in two NaDESs (choline/glycerol and choline/propylene glycol), against several drug-resistant clinical isolates and standard microbial strains has been evaluated. The main result was the inhibitory activity of the EVOO extract in choline/glycerol as well as oleacein in choline/propylene glycol toward drug-resistant Gram-positive and -negative strains. Specifically, the EVOO extract in choline/glycerol showed the highest antibacterial activity against several clinical strains of *Staphylococcus aureus*, whereas oleacein in choline/propylene glycol was the most effective toward various clinical strains of *Escherichia coli*, *Pseudomonas aeruginosa*, and *Klebsiella pneumoniae*. In addition, all the formulations tested were effective against *Candida* spp. In conclusion, our results suggest EVOO-based formulations in NaDESs as an interesting strategy that may help in reducing the risk of development of drug resistance. Under this perspective, the usage of NaDESs for the preparation of new antimicrobial formulations may represent a promising approach.

## Introduction

Antibiotic resistance is currently one of the greatest threats to human health resulting in an increased number of deaths caused by once curable infections (Huemer et al., 2020). Globally, antibiotic-resistant pathogens are responsible for approximately 700,000 deaths/year, and 10 million deaths/year are expected by 2050, a number greatly exceeding deaths from cancer (O’Neill, 2014; de Kraker et al., 2016; Aslam et al., 2018). However, the estimated number of deaths may more likely increase following the ongoing coronavirus disease 2019 (COVID-19) pandemic, characterized by elevated antibiotic use in patients infected with SARS-CoV-2 and by the exponential growth in disinfectant use (Ansari et al., 2021).

Over the years, the widespread use of antibiotics has led to an increased incidence of bacterial resistance, beginning with the emergence of methicillin-resistant *Staphylococcus aureus*, which has rapidly become the most frequently occurring resistant pathogen identified in many parts of the world, including Europe (Guo et al., 2020). Following, one recent issue is the increasing prevalence of extended-spectrum beta-lactamase (ESBL)-producing Enterobacteriaceae all over the world, further limiting treatment options (Wilson and Török, 2018). For example, ESBL-producing *E. coli* are resistant to penicillins and most cephalosporins and are often co-resistant to other antimicrobial classes, such as trimethoprim–sulfamethoxazole, quinolones, and aminoglycosides (De Oliviera et al., 2020; Kakoullis et al., 2021). Among the pathogens with growing multidrug resistance, the WHO included ESKAPE pathogens (*Enterococcus faecium*, *Staphylococcus aureus*, *Klebsiella pneumoniae*, *Acinetobacter baumannii*, *Pseudomonas aeruginosa*, and *Enterobacter* species) against which new antibiotics are urgently needed (World Health Organization, 2017; De Oliveira et al., 2020; Ma et al., 2020).

In this scenario, currently available antibiotic treatments often have limited or no efficacy, and novel therapeutic approaches need to be investigated. Efforts are now focusing on natural products, considered extremely interesting and promising for the preparation of pharmacological formulations and nutraceuticals (Sessa et al., 2015; Sharifi-Rad et al., 2020; Okagu et al., 2021; Oliveira et al., 2021). Recently, the interest in nutraceuticals is growing as most of them possess multiple therapeutic properties, including antimicrobial, antioxidant, anti-inflammatory, and anticancer activities (Di Pietro et al., 2013; Sessa et al., 2017a; Sessa et al., 2017b; Filardo et al., 2020; Mattioli et al., 2020; Ayatollahi et al., 2021). In this regard, extra virgin olive oil (EVOO) obtained from cold pressure of *Olea europaea* L. drupes, a cornerstone in the Mediterranean diet, is well known for its nutritional properties and health effects, especially for the prevention of cardiovascular diseases and metabolic disorders. In fact, consumption of olive oil is able to reduce lipid and DNA oxidation; ameliorate lipid profile and insulin resistance, endothelial dysfunction, and inflammation; and lower blood pressure in hypertensive patients (De Santis et al., 2019; Romani et al., 2019; Serreli and Deiana, 2020).

Traditionally, health properties have been largely attributed to the high concentration of monounsaturated fatty acids (98–99% of the total weight of EVOO); however, in recent years, beneficial effects have also been related to other components, particularly polyphenols with promising antimicrobial properties. Specifically, hydroxytyrosol, tyrosol, oleuropein, and several EVOO extracts have been shown to have antibacterial activity against several oral and foodborne pathogens, as well as against some pathogens responsible for hospital and community infections (Medina et al., 2006; Karaosmanoglu et al., 2010; Nazzaro et al., 2019). Other EVOO compounds, such as the secoiridoids oleocanthal and oleacein, belonging to polyphenols, are nowadays two of the most interesting bioactive natural products under investigation. Both oleocanthal and oleacein have been described to exert anti-inflammatory effects, selective cytotoxicity for cancer cells, and promising neuroprotective activity (Pang and Chin 2018; Lonzano-Castellón et al., 2020; Emma et al., 2021; Gabbia et al., 2021). Interestingly, their antibacterial effects toward several oral pathogens and a *Candida albicans* strain have recently been described (Karygianni et al., 2019).

Despite promising pharmacological activities, oleocanthal and oleacein cannot be easily obtained *via* chemical synthesis, and their isolation and purification from EVOO are expensive with low yields. Recently, a green route for obtaining high yields of oleocanthal and oleacein from EVOO, *via* natural solvents, namely, natural deep eutectic solvents (NaDESs), emerging as powerful and biocompatible solvents, has been proposed (Francioso et al., 2020). Indeed, this is an excellent means for complete dissolution and extraction of a wide range of non-polar and polar compounds.

Here, we evaluated, for the first time, the antimicrobial activity of different green extra virgin olive oil-based formulations in NaDESs. Specifically, the antimicrobial activity of the EVOO extract, as well as purified oleocanthal and oleacein in NaDESs against several drug-resistant clinical isolates and standard microbial strains, has been evaluated.

## Materials and Methods

### Antimicrobial Agents

Extra virgin olive oil (EVOO) (*Olea europaea* L., Coratina cultivar) was obtained from a local market in the Puglia region (Italy). The polyphenolic fraction was extracted as described in Francioso et al. (2020). Briefly, NaDESs were prepared by mixing two components (choline:glycerol 1:1.5 molar ratio and choline:propylene glycol 1:3.3 molar ratio) at 70°C under magnetic stirring for 1 h. After cooling, EVOO was added to the NaDESs in the ratio of 1:20 v/v (NaDES:EVOO). The extraction was carried out under magnetic stirring at room temperature for 15 min and then transferred *via* a separatory funnel for decantation and phases separation. The extract was analyzed to determine the total polyphenol content and polyphenolic composition.

In addition, two pure compounds purified from EVOO (following the method described in Francioso et al., 2020, provided by Active-Italia srl), namely, oleocanthal and oleacein, were dissolved in NaDES (choline:glycerol, 1:1.5 molar ratio; choline:propylene glycol, 1:1.5 molar ratio) or in methanol and tested for their antimicrobial activity.

### Determination of Total Phenols

Total phenols were determined by the Folin–Ciocalteu assay as described by Singleton and Rossi (1965). Briefly, the reaction solution was prepared by mixing 790 μl of distilled water with 10 μl of standard, sample, or blank. To these, 50 μl of Folin–Ciocalteu reagent (Merck KGaA, Darmstadt, Germania) was added, incubated for 3 min at room temperature, and then 150 μl of 20% (w/v) Na_2_CO_3_ was added. After 2 h of incubation, the absorbance at 760 nm was measured using a Hitachi U2000 spectrophotometer (Hitachi, Tokyo, Japan). The results were expressed as gallic acid equivalents (GAE).

### Polyphenolic Analysis by UPLC-DAD/MS

Polyphenolic analysis of the extracts was performed on a Waters Acquity H-Class UPLC system, as previously described by Francioso et al. (2020). The chromatographic system was coupled to a photodiode array and a single-quadruple mass detector with an electrospray ionization source. Chromatography was performed on a reverse-phase C18 column (Phenomenex Kinetex, 100 mm × 2.1 mm i.d., 2.6 μm particle size). Solvent A was 0.1% aqueous formic acid (Merck), and solvent B was 0.1% formic acid in Methanol (UPLC gradient grade, Merck). The flow rate was 0.5 ml/min, and the column temperature was set at 35°C. Elution was performed with a linear gradient from 2 to 100% B in a total time of 17 min including re-equilibration. The samples were diluted in the mobile phase and injected through the needle. The photodiode array detector was set up in the range of 200–600 nm. Mass spectrometric detection was performed in the negative electrospray ionization mode, using nitrogen as the nebulizer gas. Analyses were performed in the Total Ion Current (TIC) mode with a mass range of 50–1000 m/z. The capillary voltage was 0.8 kV, cone voltage 15 V, ion source temperature 120°C, and probe temperature 600°C. Compounds were identified by retention time, m/z, UV-Vis spectrum, and comparing them with commercially available standards (obtained from Merck). Quantification of each compound was performed by using standard calibration curves in the range of 0.1–2 nmol.

### Microbial Strains and Cultures

The antimicrobial activity was investigated against a representative range of standard bacterial strains and drug-resistant bacterial clinical isolates. Specifically, standard strains included Gram-positive and Gram-negative bacteria [*Staphylococcus aureus* (*S. aureus*) ATCC 6538, *Pseudomonas aeruginosa* (*P. aeruginosa*) ATCC 15442, *Escherichia coli* (*E. coli*) ATCC 10536, *Salmonella choleraesuis* (*S. choleraesuis*) ATCC 10708]. Resistant bacterial strains included clinical isolates of *S. aureus*, *P. aeruginosa*, and *β*-lactamase (ESBL)-producing strains such as *E. coli* and *Klebsiella pneumoniae* (*K. pneumoniae*). Last, yeasts such as *Candida albicans* (*C. albicans*) ATCC 10231 and a clinical strain of *Candida parapsilosis* (*C. parapsilosis*) were examined. Clinical strains were isolated, at the Microbiology Unit of the University Hospital in Rome, from samples processed during routine analysis and cultured in accordance with guidelines approved by the management of the hospital for routine care purposes. Then, all isolates were identified by matrix-assisted laser desorption ionization–time-of-flight mass spectrometry (MALDI-TOF, Bruker, Bremen, Germany). Antimicrobial susceptibility testing was performed by using a VITEK 2 System (bioMérieux, Inc. France) and by a MicroScan WalkAway System 96 Plus (Beckman Coulter S.r.l.), by using antimicrobial panels provided by the manufacturer for Gram-negative (Vitek 2 AST-N397; Microscan NMDRM1) and Gram-positive (Vitek 2 AST-P592; Microscan PM-STA36) bacteria. Results were interpreted according to the EUCAST clinical breakpoints (https://www.eucast.org/fileadmin/src/media/PDFs/EUCAST_files/Breakpoint_tables/v_12.0_Breakpoint_Tables.pdf). *S. aureus* ATCC 25923, *E. coli* ATCC 25922, and *P. aeruginosa* ATCC 27853 were used as quality control strains.

The source and resistance profile of the microbial strains are described in Table 1. According to the European Centre for Disease Prevention and Control (ECDC), multi-drug resistance is defined as a resistance to at least one agent in three or more antimicrobial categories (Magiorakos et al., 2012).

**TABLE 1:** Antibiotic resistance profile of microbial strains.

Strain	Source	Antibiotic resistance
*S. aureus 1*	Rectal swab	BZP
*S. aureus 2*	Catheter	BZP, OXA, CLIN, ERT, LEV, RIF
*S. aureus 3*	Skin swab	BZP, OXA, CLIN, ERT, LEV
*S. aureus* ATCC 6538	American Type Culture Collection	Sensitive
*E. coli 1*	Urine	AMC
*E. coli 2*	Tracheal aspirate	β-Lactamase-producing strain
		AMC, FEP, CAZ, CIP, P/T, TOB, G; CTX, TMP-SMX
*E. coli* ATCC 10536	American Type Culture Collection	Sensitive
*P. aeruginosa 1*	Bronchoalveolar lavage fluid	AK, FEP, CAZ, CIP, IPM, MEM, TOB, P/T, CZA
*P. aeruginosa 2*	Skin swab	FEP, CAZ, CIP, IPM, P/T
*P. aeruginosa* ATCC 15442	American Type Culture Collection	Sensitive
*K. pneumoniae*	Urine	β-Lactamase-producing strain
		AK, AMC, CTX, FEP, CAZ, CZA, CIP, IPM, MEM, P/T, TOB, FOS, G
*S. choleraesuis* ATCC 10708	American Type Culture Collection	Sensitive
*C. parapsilosis*	Tracheobronchol aspirate	Sensitive
*C. albicans* ATCC 10231	American Type Culture Collection	Sensitive

BZP, benzylpenicillin; OXA, oxacillin; CLIN, clindamycin; ERT, erythromycin; LEV, levofloxacin; RIF, rifampicin; AMC, amoxicillin/clavulanic acid; AK, amikacin; FEP, cefepime; CAZ, ceftazidime; CIP, ciprofloxacin; IPM, imipenem; MEM, meropenem; TOB, tobramycin; P/T, piperacillin–tazobactam; CZA, ceftazidime–avibactam; G, gentamicin, FOS, fosfomycin; CTX, cefotaxime; TMP-SMX, trimethoprim–sulfamethoxazole.

Bacterial strains and fungal strains were streaked from stock cultures stored at −80°C onto tryptic soy agar (TSA) and sabouraud dextrose agar (SDA), and incubated, for 24 h, at 37°C and 35°C, respectively.

### Antibacterial Susceptibility Testing

The minimum inhibitory concentration (MIC) of the extracts and the pure compounds was determined using microdilution assay in Muller–Hinton (MH) broth in accordance with the Clinical and Laboratory Standards Institute guidelines (CLSI, 2019). Briefly, bacterial inocula were prepared by suspending colonies into MHB from 18 to 24 h on TSA plates and standardized to 1 × 10^8^ colony-forming units (CFU)/ml using a photometric device (optical density at 620 nm). Then, bacterial suspensions were further diluted at 1:20 in MHB to yield a bacterial cell density of 5 × 10^6^ CFU/ml.

In 96-well flat-bottom microtiter plates, 100 µL of serial dilutions of the antimicrobial agents were inoculated with 10 µL of inoculum (final bacterial concentration 5 × 10^5^ CFU/ml). Serial two-fold dilutions of EVOO extracts in choline/glycerol and choline/propylene glycol were prepared using MHB with polyphenol concentrations from 7200 μg/ml to 225 μg/ml, and from 4680 μg/ml to 585 μg/ml, respectively.

The concentrations of oleocanthal and oleacein in choline/glycerol were twofold serial dilutions ranging from 5.056 μg/ml to 79 μg/ml and from 3.376 μg/ml to 53 μg/ml, respectively. For oleocanthal and oleacein in choline/propylene glycol, the concentrations ranging from 4528 μg/ml to 71 μg/ml and from 3.232 μg/ml to 50 μg/ml, respectively, were used. Last, for oleocanthal and oleacein in methanol, the concentrations ranged from 9.080 μg/ml to 71 μg/ml and from 6456 μg/ml to 50 μg/ml, respectively.

At the same time, a dilution series of NaDESs (choline/glycerol; choline/propylene glycol) was assayed in order to exclude their potential inhibitory effects.

The MIC value was determined as the lowest concentration of extracts or pure compounds able to inhibit the visible growth of each bacterial strain after 24-h incubation at 37°C (CLSI, 2019). To determine the minimum bactericidal concentration (MBC), 10 µL were collected from each well that contained no bacterial growth and was subcultured on agar plates (Muller–Hinton agar); the lowest concentration capable of inhibiting bacterial growth on agar surface was considered as MBC (Krishnamoorthy et al., 2018; Wijesundara et al. 2021)**.**

In every set of experiments, a positive control (gentamicin or oxacillin 16 μg/ml), negative vehicle control (antimicrobial agent and MHB), and culture control (MHB and bacteria only) were included.

### Antifungal Susceptibility Testing

Minimum inhibitory concentrations (MIC) and minimum fungicidal concentrations (MFC) were determined using the broth micro-dilution method according to Clinical Laboratory Standards Institute (CLSI) document M-27A3 (CLSI, 2008). Briefly, fungal inocula were prepared by suspending colonies into NaCl 0.5% from 24 h on SDA plates. The suspended cultures were diluted with RPMI 1640 to a cell density of 1-5 x 10^6^ CFU/ml adjusted based on the optical density at 530 nm.

In 96-well flat-bottom microtiter plates, 100 µL of tested antimicrobial dilutions were inoculated with 100 µL of inoculum (final concentration 5 × 10^2^–2.5 × 10^3^ CFU/ml).

The MIC value was determined as the lowest concentration of the extracts or the pure compounds that were able to inhibit the microorganism growth after 24 h of incubation at 35°C. To determine the minimum fungicidal concentration (MFC), 10 µL sample collected from each well without visible growth were subcultured onto agar plates; the lowest concentration capable of inhibiting fungal growth on agar surface was considered as the MFC.

In every set of experiments, a positive control (fluconazole 64 μg/ml), a negative vehicle control (antimicrobial agent and RPMI), and a cultured control (RPMI and bacteria only) were included.

### Cytotoxicity Assay

The cytotoxic effect of EVOO extracts in NaDESs was evaluated using primary human keratinocytes (HEka, cat. no. C0055C, ThermoFisher Scientific, United States). Briefly, 4000 cells per well were plated in a 96-well plate, which was then incubated at 37°C, 5% CO_2_ in Dulbecco’s modified Eagle’s medium with l-glutamine and glucose supplemented with 10% heat-inactivated fetal bovine serum. Following a 24-h incubation, the cells were exposed to two-fold dilutions of the antimicrobial agents (4680 to 1170 μg/ml), with three wells for each dilution, for 24 h at 37°C. Then, the number of viable cells was assessed by MTT (3-(4,5-dimethylthiazol-2-yl)-2,5-diphenyltetrazolium bromide, a tetrazole) assay, as previously described (Mastromarino et al., 2014).

### Statistical Analysis

The OD values from the cytotoxicity assay were expressed as mean ± standard deviation (SD) of three replicates from three independent experiments. Two-way ANOVA was performed for the analysis of variance. All statistical calculations were performed in Microsoft Excel software (version 2110), by using the Real Statistics Resource Pack (release 7.9.1, https://www.real-statistics.com, accessed on 15 March 2022). A value of *p* ≤ 0.05 was considered statistically significant.

## Results

### Polyphenol Composition of Extra Virgin Olive Oil Extracts in NaDES

**FIGURE 1:**
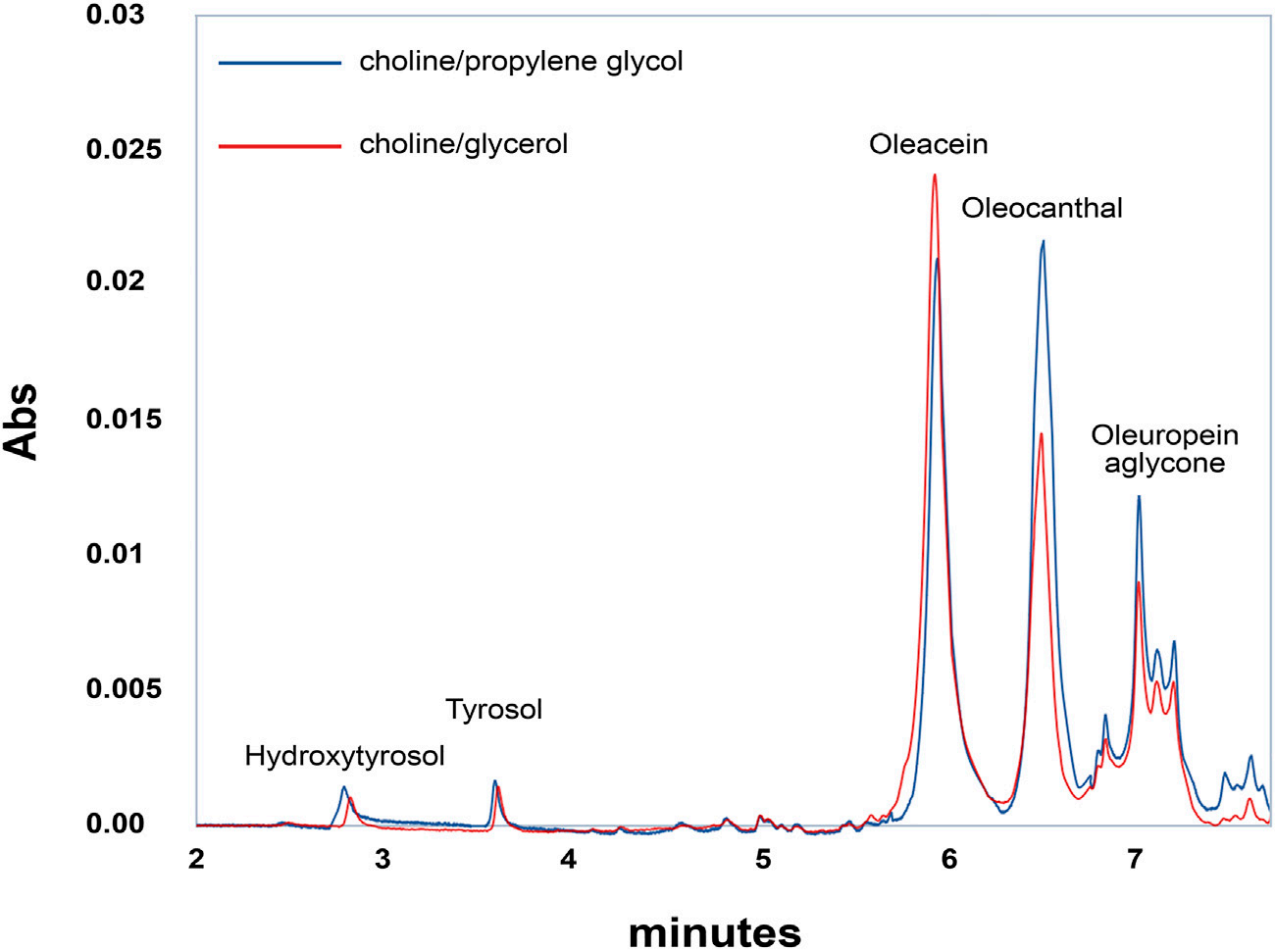
Representative chromatographic profile of a polyphenolic extract from EVOO. Blue line represents choline:glycerol extract. Red line represents choline:propylene glycol extract. EVOO extract was diluted 1:100 in water and 10 µl analyzed *via* UPLC/DAD/MS as described in Materials and Methods. Peak identification was performed on the basis of retention time, UV-Vis, and mass spectra.

EVOO extracts obtained from the Coratina cultivar are among the richest in polyphenols. Figure 1 reports the chromatographic analyses of polyphenols extracted from EVOO by two different NaDESs, choline/glycerol and choline/propylene glycol. The chromatographic profiles revealed the presence of four main components: the simple phenolic alcohols hydroxytyrosol and tyrosol, and the two secoiridoids oleacein and oleocanthal, along with oleuropein aglycone and other minor products eluted in the last part of the chromatographic gradient.

Quantitative analysis reveals that oleacein and oleocanthal are the predominant components in Coratina EVOO, largely exceeding oleuropein aglycone, tyrosol, and hydroxytyrosol. Interestingly, the NaDESs containing propylene glycol is able to extract oleocanthal more efficiently than the NaDESs containing glycerol, due to the less hydrophilic nature of propylene glycol. The polyphenol composition of EVOO extracts is reported in Table 2.

**TABLE 2:** Polyphenol composition of EVOO extracts in NaDESs.

EVOO extract	Oleocanthal (mM)	Oleacein (mM)	Polyphenols^a^ (mM)
Choline/glycerol	5.2	6.6	52.9 (9 mg/ml)
Choline/propylene glycol	9.3	6.3	68.8 (11.7 mg/ml)

a Determined by Folin–Ciocalteu assay and expressed as gallic acid equivalents.

### Antimicrobial Activity

The two EVOO extracts in NaDESs and two purified bioactive compounds, oleocanthal and oleacein, dissolved in the same NaDESs, were screened. Tables 3–8 show the MIC and MBC values for each product examined against the bacterial and fungal strains.

**TABLE 3:** Antibacterial activity of EVOO extracts in NaDESs against Gram-positive standard strains and drug-resistant clinical isolates.

Strain	EVOO extract choline/glycerol (µg/ml)^a^		EVOO extract choline/propylene glycol (µg/ml)^a^	
	MIC	MBC	MIC	MBC
*S. aureus* ATCC 6538	900	900	1170	2340
*S. aureus* 1	900	1800	1170	1170
*S. aureus* 2	900	900	1170	1170
*S. aureus* 3	900	1800	1170	2340

a Expressed as gallic acid equivalents.

**TABLE 4:** Antibacterial activity of EVOO extracts in NaDESs against Gram-negative standard strains and drug-resistant clinical isolates.

Strain	EVOO extract choline/glycerol (µg/ml)^a^		EVOO Extract choline/propylene glycol (µg/ml)^a^	
	MIC	MBC	MIC	MBC
*E. coli* ATCC 10536	1800	1800	1170	2340
*E. coli* 1	1800	3600	2340	2340
*E. coli* 2	1800	3600	2340	2340
*P. aeruginosa* ATCC 15442	1800	3600	1170	2340
*P. aeruginosa 1*	1800	1800	1170	2340
*P aeruginosa 2*	1800	1800	2340	2340
*S. choleraesuis* ATCC 10708	1800	3600	2340	2340
*K. pneumoniae*	1800	3600	2340	2340

a Expressed as gallic acid equivalents.

**TABLE 5:** Antifungal activity (µg/ml) of EVOO extracts in NaDESs against *Candida* spp.

Strain	EVOO extract choline/glycerol (µg/ml)^a^		EVOO extract choline/propylene Glycol (µg/ml)^a^	
	MIC	MFC	MIC	MFC
*C. albicans* ATCC 10231	225	450	292	585
*C. parapsilosis*	225	900	292	585

a Expressed as gallic acid equivalents.

**TABLE 6:** Antibacterial activity of oleocanthal and oleacein in NaDESs against Gram-positive standard strains and drug-resistant clinical isolates.

Strain	Oleocanthal choline/glycerol (µg/ml)		Oleocanthal choline/propylene glycol (µg/ml)		Oleacein choline/glycerol (µg/ml)		Oleacein choline/propylene glycol (µg/ml)	
	MIC	MBC	MIC	MBC	MIC	MBC	MIC	MBC
*S. aureus* ATCC 6538	316	632	283	566	211	422	404	808
*S. aureus* 1	632	1264	1132	1132	1688	1688	808	1616
*S. aureus* 2	1264	2528	1132	2264	1688	1688	1616	1616
*S. aureus* 3	1264	2528	1132	1132	1688	1688	808	1616

**TABLE 7:** Antibacterial activity of oleocanthal and oleacein in NaDESs against Gram-negative standard strains and drug-resistant clinical isolates.

Strain	Oleocanthal choline/glycerol (µg/ml)		Oleocanthal choline/propylene glycol (µg/ml)		Oleacein choline/glycerol (µg/ml)		Oleacein choline/propylene glycol (µg/ml)	
	MIC	MBC	MIC	MBC	MIC	MBC	MIC	MBC
*E. coli* ATCC 10536	1264	2528	1132	1132	1688	1688	808	1616
*E. coli* 1	632	632	566	1132	422	844	404	808
*E. coli* 2	1264	1264	1132	1132	844	844	202	404
*P. aeruginosa* ATCC 15442	1264	2528	1132	2264	1688	1688	808	1616
*P. aeruginosa 1*	632	1264	566	1132	844	844	404	404
*P aeruginosa 2*	632	632	566	1132	422	422	404	404
*S. choleraesuis* ATCC 10708	316	632	283	1132	211	422	808	1616
*K. pneumoniae*	316	632	283	1132	422	422	404	808

**TABLE 8:** Antifungal activity of oleocanthal and oleacein in NaDES against *Candida* spp.

Strain	Oleocanthal choline/glycerol (µg/ml)		Oleocanthal choline/propylene glycol (µg/ml)		Oleacein choline/glycerol (µg/ml)		Oleacein choline/propylene glycol (µg/ml	
	MIC	MFC	MIC	MFC	MIC	MFC	MIC	MFC
*C. albicans* ATCC 10231	158	632	283	566	211	422	202	404
*C. parapsilosis*	158	1264	283	1132	211	844	202	808

#### Antibacterial Activity of Extra Virgin Olive Oil Extracts

As shown in Tables 3, 4, EVOO extracts, both in choline/glycerol and choline/propylene glycol, had antibacterial activity against Gram-positive and Gram-negative bacteria including standard strains and drug-resistant clinical isolates. Overall, Gram-positive bacteria (MIC 900-1170 μg/ml, MBC 900-2340 μg/ml) were more susceptible to EVOO extracts than Gram-negative bacteria (MIC 1170-2340 μg/ml, MBC 1800-3600 μg/ml). In particular, the antibacterial activity of the EVOO extract in choline/glycerol (MIC 900 μg/ml; MBC 900-1800 μg/ml) toward Gram-positive bacteria was higher than that of the EVOO extract in choline/propylene glycol (MIC 1170 μg/ml; MBC 1170-2340 μg/ml). For most of the Gram-negative bacteria, the lower MIC value was observed for the EVOO extract in choline/glycerol, whereas the lower MBC value was observed for EVOO extract in choline/propylene glycol.

Interestingly, among the multidrug-resistant strains, the most susceptible bacteria to the EVOO extract in choline/glycerol were *S. aureus* strain 2 (MIC/MBC 900 μg/ml) and *P. aeruginosa* strains 1 and 2 (MIC/MBC 1800 μg/ml). Similarly, the most susceptible bacteria to the EVOO extract in choline/propylene glycol were *S. aureus* strain 2 (MIC/MBC 1170 μg/ml) and *P. aeruginosa* strain 1 (MIC 1170 μg/ml, MBC 2340 μg/ml). More interestingly, ESBL-producing and carbapenem-resistant *K. pneumoniae* as well as ESBL-producing *E. coli* strain 2, resistant to several antibiotic classes (MIC 1800 μg/ml), were also susceptible to the EVOO extract in choline/glycerol, whereas the MBC value against both strains was the lowest for the EVOO extract in choline/propylene glycol (MBC 2340 μg/ml).

#### Antifungal Activity of EVOO Extracts

EVOO extracts, both in choline/glycerol and choline/propylene glycol, had antifungal activity against *C. albicans* ATCC 10231 and clinical isolate of *C. parapsilosis* with MIC and MFC values of 225–292 μg/ml and 450-900 μg/ml, respectively (Table 5). Overall, EVOO extracts in choline/glycerol (MIC 225 μg/ml) and in choline/propylene glycol (MIC 292 μg/ml) showed a similar inhibitory activity toward *C. albicans* and *C. parapsilosis*. On the contrary, the MFC value of the EVOO extract in choline/propylene glycol (MFC 585 μg/ml) was lower than that in the EVOO extract in choline/glycerol (MFC 450-900 μg/ml).

#### Antibacterial Activity of Oleocanthal and Oleacein

As shown in Tables 6, 7, oleocanthal and oleacein, both in choline/glycerol and choline/propylene glycol, had antibacterial activity against Gram-positive and Gram-negative bacteria including standard strains and drug-resistant clinical isolates. Overall, oleacein in choline/propylene glycol displayed the highest antibacterial activity against Gram-negative bacteria, with MIC and MBC ranging from 202 μg/ml to 808 μg/ml, and from 404 μg/ml to 1616 μg/ml, respectively. Specifically, the highest susceptibility was observed for multidrug-resistant ESBL-producing *E. coli* (strain 2) (MIC 202 μg/ml and MBC 404 μg/ml), as well as *P. aeruginosa* strains 1 and 2 (MIC/MBC 404 μg/ml). Interestingly, oleacein in choline/propylene glycol was also active against Gram-positive bacteria, with a MIC range of 404 μg/ml to 808 μg/ml, apart from *S. aureus* strain 2 (MIC 1616 μg/ml), although its MBC value was lower than that of oleacein in choline/propylene glycol.

Last, higher MIC and MBC values for oleocanthal and oleacein in methanol were observed toward standard strains of *E. coli* and *P. aeruginosa*, than the same compounds dissolved in the two NaDESs (Supplementary Table S1).

#### Antifungal Activity of Oleocanthal and Oleacein

Oleocanthal and oleacein, both in choline/glycerol and choline/propylene glycol, had antifungal activity against *C. albicans* ATCC 10231 and clinical isolate of *C. parapsilosis* (Table 8). Overall, oleocanthal (MIC 158-283 μg/ml) and oleacein (MIC 202-211 μg/ml) showed a similar inhibitory activity toward *C. albicans* and *C. parapsilosis*. On the contrary, the MFC value of oleacein (MFC 404-844 μg/ml) was lower than that of oleocanthal (MFC 566 -1264 μg/ml).

More importantly, oleacein in choline/propylene glycol was the most effective toward *C. albicans* and *C. parapsilosis* with MIC values of 202 μg/ml for both strains and MFC values of 404 μg/ml and 808 μg/ml, respectively.

#### Antimicrobial and Antifungal Activity of NaDESs

To assess the potential inhibitory effects of choline/glycerol and choline/propylene glycol against all microbial strains examined, NaDES concentrations ranging from 25 to 0.77% (v/v) were also investigated. Both choline/glycerol and choline/propylene glycol did not show antimicrobial or antifungal activity at or below a concentration of 25% (v/v).

### Cytotoxic Activity

In order to assess the cytotoxic effect of EVOO extracts in NaDESs as well as of oleacein and oleocanthal in NaDES, HEKa cells were incubated with increasing concentrations of each product for 24 h, and then cell viability was measured by the MTT assay. No significant cytotoxicity was observed at the concentrations that showed antimicrobial activities (Supplementary Figure S1).

## Discussion

To our knowledge, this is the first study evaluating the antimicrobial activity of EVOO extracts using as two natural solvents NaDESs (choline/glycerol and choline/propylene glycol). In addition, its main components, oleacein and oleocanthal in choline/glycerol and choline/propylene glycol, have also been investigated. The main result of clinical importance was the inhibitory activity of the EVOO extract in choline/glycerol and that of oleacein in choline/propylene glycol toward drug-resistant Gram-positive and -negative bacterial strains. Specifically, the EVOO extract in choline/glycerol showed the highest antibacterial activity against several clinical strains of *S. aureus*, whereas oleacein in choline/propylene glycol was the most effective toward various clinical strains of *E. coli*, *P. aeruginosa* and *K. pneumoniae*.

The highest inhibitory effect of the EVOO extract in choline/glycerol against drug-resistant strains of *S. aureus* may be attributed to the synergistic effect of its main components as detected by UPLC/DAD/MS analysis (oleacein, oleocanthal, and oleuropein aglycone), since, in our study, both oleacein and oleocanthal alone were less active. Also, Karygianni et al. (2019) demonstrated low antibacterial activity of oleacein, oleocanthal, and oleuropein alone toward *S. aureus*, further suggesting that the combination of the components present in our EVOO extract might possess a higher effect.

Of note, the EVOO extract in choline/glycerol was also active toward different drug-resistant Gram-negative strains including *P. aeruginosa*, *E. coli*, and *K. pneumoniae*. Such effects may be due to the ability of choline/glycerol to extract more hydrophilic compounds from EVOO, such as oleacein, that might be more effective against Gram-negative bacteria since their outer membrane surrounding the cell wall has been described to hinder the diffusion of non-polar molecules through the lipopolysaccharide (Vaara, 1992; Denyer and Maillard, 2002).

Even more important is the highest inhibitory effect of oleacein in choline/propylene glycol toward all the multidrug-resistant Gram-negative strains examined, including the ESKAPE pathogens such as *P. aeruginosa* and *K. pneumoniae*. ESKAPE pathogens have been recognized as a considerable public health concern because their resistance has reached alarming levels, requiring urgent intervention as underlined by the most recent WHO reports (World Health Organization, 2017). ESKAPE pathogens are usually encountered in the hospital setting and may become, in the future, a serious threat in community settings where the overuse and misuse of antimicrobial agents are increasing.

The highest antibacterial activity of oleacein in choline/propylene glycol toward multidrug-resistant Gram-negative bacteria may also be due to its NaDES content (2.1–8.4% v/v) since our preliminary experiments showed a lower activity of oleacein in methanol. Such effect is also suggested by evidence in the literature, where oleacein in another solvent (1.1% of DMSO) has been demonstrated to be less active toward Gram-negative strains (Karygianni et al., 2019). A potential mechanism through which choline/propylene glycol may augment the antibacterial effect of oleacein might consist in increased membrane permeability and/or damaged morphology of bacterial cells, facilitating the uptake of the active compound. Indeed, many studies demonstrated that NaDESs significantly increase the bioavailability of bioactive molecules and drugs (Faggian et al., 2016; Sut et al., 2017; Jeliński et al., 2019; da Silva et al., 2021; Mustafa et al., 2021).

In addition to improving the antimicrobial activity, the usage of NaDESs also has additional advantages; NaDESs are characterized by a low environmental impact, combined with the optimization of the extraction of active principles. Indeed, toxic by-products or residual solvents are not generated (Francioso et al., 2020), as also supported by the absence of cytotoxic effects found in our study.

Another interesting finding observed in our study is the marked antifungal activity of all the assayed compounds, including EVOO extract, oleacein as well as oleocanthal in both NaDESs, and *Candida* spp. To date, available antifungal drugs are few, and their widespread use has led to the development of drug resistance in the treatment of *Candida* infections, a problem of growing importance, especially in immunodeficient conditions (Pristov and Ghannoum, 2019).

Overall, our results are another interesting piece of the EVOO puzzle and, mainly, of its antimicrobial properties. Indeed, in the literature, there are mentions of the antimicrobial activity of olive oil extracts on numerous standard microorganisms including *S. aureus*, *E. coli*, *P. aeruginosa*, *C. albicans*, foodborne pathogens such as, *Listeria* spp., *Salmonella* spp. and several clinical isolates such as *Helicobacter pylori*, *Shigella sonnei*, *Bacteroides* spp*.*, and *Yersinia* spp. (Medina et al., 2006; Romero et al., 2007; Medina et al., 2009; Fei et al., 2018, 2019; Guo et al., 2019; Nazzaro et al., 2019; Guo et al., 2020). Nevertheless, a comparison of the antibacterial activity observed among several studies is quite difficult since different experimental protocols and a diverse variety of olive plants and different types of olive oil (olive oil, virgin olive oil, EVOO), were used. Regarding the chemical composition, it is well known that it depends on different factors such as cultivar, geographic origin, climatic conditions, and processing techniques (De Santis et al., 2019). In addition, the type of bioactive compounds of olive oil extracts is influenced by the chemical properties of the extraction solvent, as evidenced by our study where an EVOO extract rich in oleocanthal and oleacein was obtained by using NaDES.

Oleocanthal and oleacein have already been described to have antimicrobial activity against several standards strains such as *E. coli*, *S. aureus*, *L. monocytogenes*, *H. pylori*, *Pseudomonas fluorescens*, *Enterococcus faecalis*, oral streptococci, and anaerobic pathogenic bacteria as well as against *C. albicans* (Medina et al., 2006; Romero et al., 2007; Medina et al., 2009; Karygianni et al., 2019). In our study, similar MIC values of oleocanthal and oleacein for *E. coli* and lower MIC values for *S. aureus* and *C. albicans* were observed as compared to data of Karygianni et al. (2019), which used the same method for evaluating the antimicrobial activity*.*

The strength of our study lies in the investigation of several drug-resistant strains, which might better characterize the potential antibacterial activity of EVOO, oleocanthal and oleacein. However, the lack of data regarding the mechanisms by which these natural compounds may inhibit the growth of clinical isolates or cause bacterial cell death represents the main limitations of our study. However, Guo et al. (2019, 2020) found that the antibacterial effect of the olive oil polyphenol extract against several foodborne pathogens such as *L. monocytogenes*, *S. aureus*, and *S. typhimurium* was related to lower intracellular adenosine 5′-triphosphate, cell depolarization, decrease in bacterial protein and DNA, and cell fluid leakage due to destruction of cell morphology.

In conclusion, our study shows that the EVOO extract in choline/glycerol and oleacein in choline/propylene glycol have potential activity against multidrug-resistant *S. aureus* and several ESKAPE pathogens. In the future, given their broad antimicrobial activity, EVOO-based formulations in NaDES might be an interesting strategy that may help in reducing the risk of development of drug resistance. Under this perspective, the usage of NaDES for the preparation of new antimicrobial formulations may represent a new approach that deserves further research.

## Data Availability

The original contributions presented in the study are included in the article/Supplementary Material, further inquiries can be directed to the corresponding author.
