# Supplementary material for: Extra Virgin Olive Oil-Based Green Formulations With Promising Antimicrobial Activity Against Drug-Resistant Isolates
Source: Front Pharmacol. 2022 Apr 25;13:885735. doi: 10.3389/fphar.2022.885735 (PMC9082028; doi:10.3389/fphar.2022.885735)
Supplement: Supplementary file 1 [file Table1.DOCX]

**Supplementary Table S1.** Antibacterial activity of oleocanthal and oleacein in methanol against Gram-negative standard bacterial strains

| Strains | Oleocanthal/MeOH  (µg/ml) | | |  | Oleacein/MeOH  (µg/ml) | | | |
| --- | --- | --- | --- | --- | --- | --- | --- | --- |
|  | MIC |  | MBC |  | | MIC |  | MBC |
| *E. coli ATCC* 10536 | 2270 |  | 4540 |  | | 1614 |  | 3228 |
| *P. aeruginosa* ATCC 15442 | 2270 |  | 2270 |  | | 1614 |  | 1614 |

MeOH, methanol
