# Supplementary figures and images for: Extra Virgin Olive Oil-Based Green Formulations With Promising Antimicrobial Activity Against Drug-Resistant Isolates
Source: Front Pharmacol. 2022 Apr 25;13:885735. doi: 10.3389/fphar.2022.885735 (PMC9082028; doi:10.3389/fphar.2022.885735)

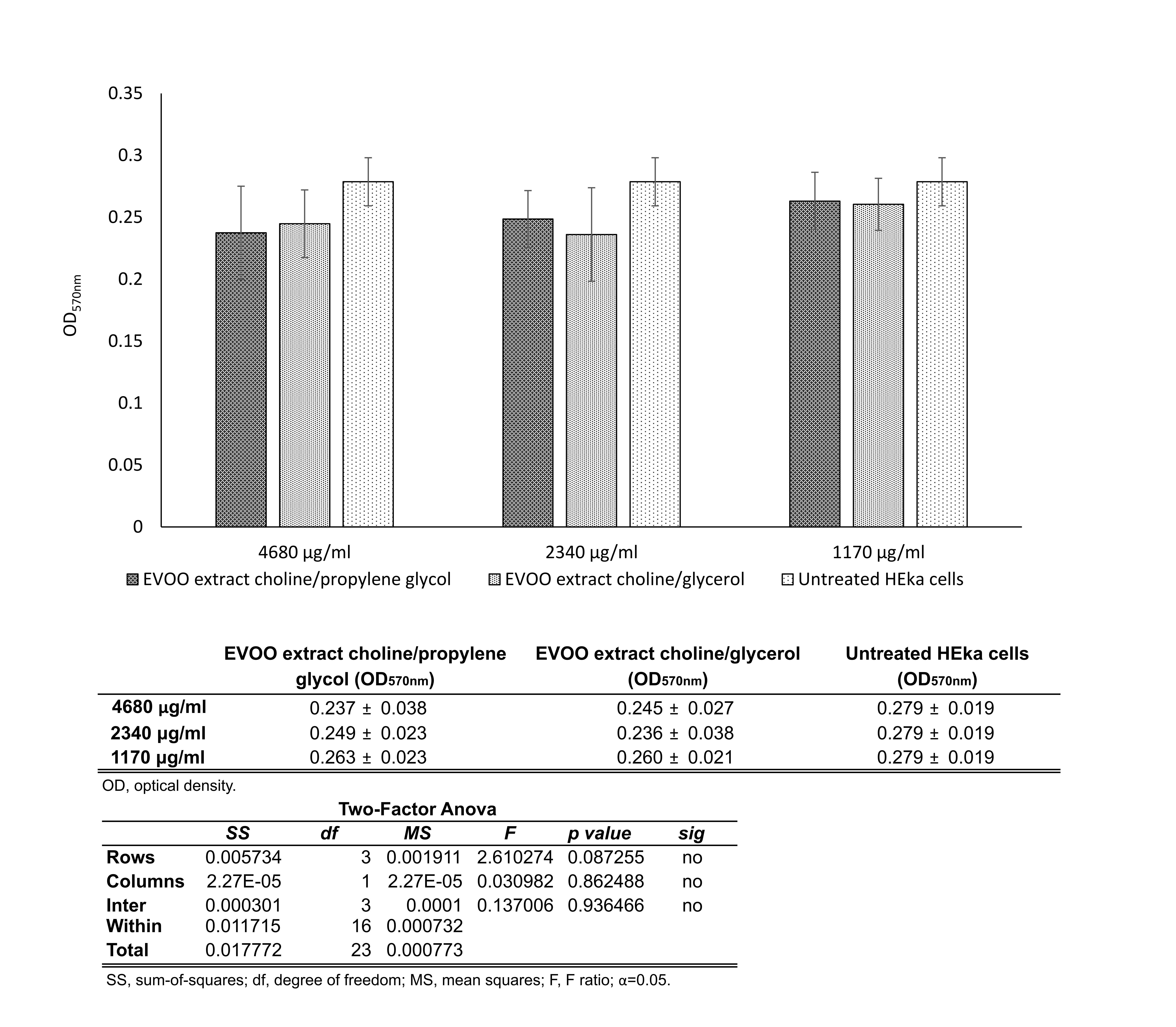

Supplement: Supplementary file 2 [file Image1.TIF]
